# Oral ingestion of hexavalent chromium through drinking water and cancer mortality in an industrial area of Greece - An ecological study

**DOI:** 10.1186/1476-069X-10-50

**Published:** 2011-05-24

**Authors:** Athena Linos, Athanassios Petralias, Costas A Christophi, Eleni Christoforidou, Paraskevi Kouroutou, Melina Stoltidis, Afroditi Veloudaki, Evangelia Tzala, Konstantinos C Makris, Margaret R Karagas

**Affiliations:** 1 Department of Hygiene, Epidemiology and Medical Statistics, Medical School, National and Kapodistrian University of Athens, 75 Mikras Asias str., Athens, 11527, Greece; 2 Institute of Preventive Medicine, Environmental & Occupational Health, Prolepsis, 7 Fragoklisias str., Maroussi, 15125, Greece; 3 Department of Statistics, Athens University of Economics and Business, 76 Patission str., Athens, 10434, Greece; 4 Cyprus International Institute for Environmental and Public Health in association with Harvard School of Public Health, Cyprus University of Technology, Eirinis 95 str., Limassol, 3041, Cyprus; 5 Department of Environmental Health, Harvard School of Public Health, 401 Park Drive str., Boston, MA, 02215, USA; 6 The Biostatistics Center, George Washington University, 6110 Executive Boulevard, Rockville, MD, 20852, USA; 7 Hellenic Cancer Registry, Hellenic Centre for Disease Control & Prevention, 3-5 Agrafon str., Maroussi, 15123, Greece; 8 Department of Community and Family Medicine, Section of Biostatistics & Epidemiology, Dartmouth Medical School, 1 Medical Center Drive, Hanover, NH, 03756, USA

## Abstract

**Background:**

Hexavalent chromium is a known carcinogen when inhaled, but its carcinogenic potential when orally ingested remains controversial. Water contaminated with hexavalent chromium is a worldwide problem, making this a question of significant public health importance.

**Methods:**

We conducted an ecological mortality study within the Oinofita region of Greece, where water has been contaminated with hexavalent chromium. We calculated gender, age, and period standardized mortality ratios (SMRs) for all deaths, cancer deaths, and specific cancer types of Oinofita residents over an 11-year period (1999 - 2009), using the greater prefecture of Voiotia as the standard population.

**Results:**

A total of 474 deaths were observed. The SMR for all cause mortality was 98 (95% CI 89-107) and for all cancer mortality 114 (95% CI 94-136). The SMR for primary liver cancer was 1104 (95% CI 405-2403, p-value < 0.001). Furthermore, statistically significantly higher SMRs were identified for lung cancer (SMR = 145, 95% CI 100-203, p-value = 0.047) and cancer of the kidney and other genitourinary organs among women (SMR = 368, 95% CI 119-858, p-value = 0.025). Elevated SMRs for several other cancers were also noted (lip, oral cavity and pharynx 344, stomach 121, female breast 134, prostate 128, and leukaemias 168), but these did not reach statistical significance.

**Conclusions:**

Elevated cancer mortality in the Oinofita area of Greece supports the hypothesis of hexavalent chromium carcinogenicity via the oral ingestion pathway of exposure. Further studies are needed to determine whether this association is causal, and to establish preventive guidelines and public health recommendations.

## Background

Hexavalent chromium Cr(VI) is recognized by the World Health Organization (WHO) as a human carcinogen through inhalation [1], but there is significant debate on the carcinogenicity of hexavalent chromium when it is orally ingested. In a recent article using data from the National Toxicology Program (NTP) of the National Institutes of Health, hexavalent chromium was identified as 'likely to be a carcinogen to humans' with an estimate of the cancer potency to humans equal to 0.5 (mg/kg/day)^-1 ^[2].

At the cellular level, Cr(VI) is a highly active carcinogen [3,4]. A key issue is whether Cr(VI) ingested through the oral route, converts to trivalent Chromium Cr(III) (which does not cross the cell membrane that easily) before entering a living cell [5]. A recent study [6] revealed that rats and mice exposed to Cr(VI)-contaminated drinking water developed gastrointestinal abnormalities, including oral and intestinal tumors. An earlier study [7] also found an increased incidence of benign and malignant combined forestomach neoplasms in mice orally exposed to Cr(VI), whereas a more recent publication [8] presented a physiologically based model of chromium kinetics according to which non reduced hexavalent chromium after oral exposure could be metabolized in the red blood cells, liver, kidney and bone.

Because areas characterized by high Cr(VI) concentrations in drinking water are relatively uncommon, human epidemiologic studies are scant. One of the most cited and controversial studies analyzing the effects of oral exposure to Cr(VI) on population cancer mortality rates was conducted near a chromium smelting plant in the Liaoning Province, China [9]. Elevated mortality rates for total cancer, lung cancer, and stomach cancer were noted. These data were re-analyzed and re-evaluated by other investigators [10,11]; their re-analysis supported the conclusions of the original study [12,13]. However, a different study [14], comparing the same exposed villages to those of nearby areas, concluded that on average, the mortality rates for lung, stomach, and total cancer were not statistically different. Thus, based on ecologic studies and animal studies, one could hypothesize that several organs could be targets of chromium carcinogenicity including the liver, kidney, bladder, gastrointestinal tract, the hematopoietic system and even bone.

In order to further examine the potential effects of elevated oral exposure to hexavalent chromium, we performed an ecological mortality study in an industrial area of Greece where the water consumed by the population was contaminated with hexavalent chromium (maximum levels ranging between 41 and 156 μg/l in 2007-2009, and presumed exposure for at least 20 years). Therefore, the goal of this study was to examine the cancer mortality in an area of Greece, historically satisfying its potable needs with a Cr(VI)-contaminated aquifer.

## Methods

### Study area location and exposure

**Figure 1:**
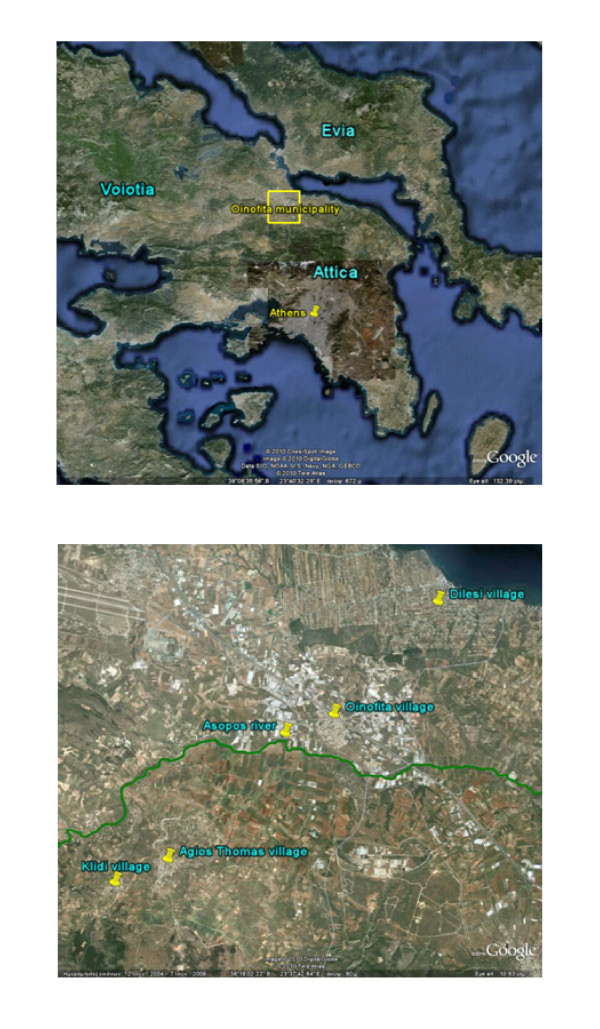
**Map of the Oinofita municipality (study area) in Greece**. Panel A: Oinofita municipality lies at the border of the Voiotia with the Attica prefecture. Panel B: Oinofita municipality is comprised of four villages: Klidi, Agios Thomas, Oinofita and Dilesi. The high industrial concentration near Asopos river can also be observed.

The Oinofita municipality (Figure 1) is situated 50 km north of Athens, Greece and is comprised of four villages that were initially rural but transformed into industrial areas in the early 1970s. In 1969, a ministerial decision gave permission for depositing processed industrial waste in the Asopos river, which runs through Oinofita. This decision, furthered by a presidential decree in 1979, permitted free disposal of processed liquid industrial waste into the river.

The Oinofita industrial region is located within Voiotia prefecture at the border of Attica prefecture (that includes the capital city of Athens). Due to the proximity to Attica, the number of industries in the Oinofita region increased precipitously after 1984, when a new law imposed restrictions on the establishment of various industries within Attica. According to the Technical Chamber of Greece [15], in 2009, there were about 700 industries operating in the Oinofita area, of which 500 generated liquid industrial waste.

Initial concerns were raised after Oinofita area citizens complained about the discoloration and turbidity of their drinking water. Regular protests ensued from the 1990s onward. In 2007, the Ministry of Environment, Regional Planning and Public Works of Greece imposed fines on 20 industries for disposing industrial waste with high levels of hexavalent chromium into the Asopos river.

Official limits on total chromium have been set by both the United States Environmental Protection Agency (EPA), equal to 100 μg/l, and the European Union (Council directive 98/83/EC), equal to 50 μg/l. However, as of yet, there are no limits set by any international body for Cr(VI). In 2009, the California Environmental Protection Agency proposed a public health goal level of 0.06 μg/l for Cr(VI) in drinking water [16].

Since 2007, three independent sets of hexavalent chromium measurements are available for the Oinofita area. These include: a) a study of the Institute of Geology and Mineral Exploration (IGME) [17] during the period November 2007 to February 2008, which detected 35 samples (out of 87) taken from different wells in the same area to have levels above 10 μg/l with a maximum value 156 μg/l; b) a study conducted by faculty of the Geology and Geo-environment department of the University of Athens [18] during the period September 2008 to December 2008, in which Cr(VI) levels ranged from 41 up to 53 μg/l in three samples taken from the public drinking water supply of Oinofita; and c) repeated measurements from the Oinofita municipality in the public drinking water supply during the period July 2007 to July 2010, in which there are 13 measurements with levels above 10 μg/l and with a maximum value of 51 μg/l. Notably all 16 measurements made in 2007 and 2008 by the Oinofita municipality, record hexavalent chromium levels above 8 μg/l (Table 1). According to official Oinofita municipality authorities, in early 2009 the main drinking water supply of Oinofita was diverted to receive water from Mornos lake (reservoir) which is part of the drinking water supply network of the city of Athens. Therefore, more recent measurements made by the Oinofita municipality (June 2009- July 2010) record relatively lower levels of Cr(VI) (<0.01-1.53 μg/l). To the best of our knowledge, there are no systematic measurements of Cr(VI) before 2007. However, a measurement made by the Oinofita municipality in 1996, showed Cr(VI) levels of 54 μg/l in the public drinking water supply.

**Table 1:** Hexavalent chromium measurements in different sites of the public drinking water supply of the Oinofita municipality during the period July 2007- June 2008

Sample	Date	Site	Level (μg/l)
1	24/7/2007	1	43

2	24/7/2007	2	51

3	24/7/2007	3	50

4	24/7/2007	4	47.9

5	24/7/2007	5	26.2

6	24/7/2007	6	27.9

7	26/10/2007	7	28

8	26/10/2007	8	10

9	8/11/2007	1	43

10	8/11/2007	9	10

11	29/11/2007	8	39

12	6/12/2007	9	11

13	6/12/2007	1	44

14	6/12/2007	10	12

15	16/6/2008	11	42.8

16	16/6/2008	12	8.3


Source: Oinofita municipality

### Study population

Using municipality records, we identified 5842 individuals who met the following criteria: a) being a legally registered citizen of the municipality at any time during the follow up period (1/1/1999 - 31/12/2009) and b) being registered as a permanent resident of Oinofita in the municipality records. The date 1/1/1999 was selected due to the fact that the municipality's vital statistics department maintained death certificates in a systematic way only after 1/1/1999.

In order to identify these individuals, we first obtained electronic records of all persons ever (since 1855) legally registered in the municipality. Greek municipality records are maintained in family units. A person remains in his/her family record from birth until marriage or until registering in another municipality. At the time of marriage a new record is created for the couple, and their children are included in that same record. In case of divorce, two new records are created and children may be included in either of the parents' records. This way each individual may have multiple records based on whether he/she has been married, divorced, or changed municipalities through the years. Furthermore, in Greece one can move into a new place of residence without registering into the new municipality, but rather maintaining his/her initial municipality record. Thus, it was essential for our study design to exclude individuals registered in the Oinofita municipality but living outside the municipality. The municipality records provide the information of whether a person is a permanent resident or has moved outside the municipality. Thus we selected persons fulfilling both criteria "a" and "b" to include in the study design. The original file with municipality records of Oinofita contained 13,582 records referring to a total of 8872 individuals. We merged the information of all records on each person into a single record per individual for the analysis.

Of the potentially eligible 8872 persons identified, 1958 did not meet the criterion of being registered citizens of Oinofita municipality at any time between 1/1/1999-31/12/2009, whereas an additional 1072 were excluded because they did not meet the residence criterion. Therefore, the resulting cohort was comprised of 5842 individuals.

The beginning of follow up period for each individual was set as either a) January 1, 1999 for individuals registered in the municipality before this date, or b) the date of registration in the municipality for those registered after January 1, 1999. The end of the follow up was set as either a) the date of death or the date of deletion from the records because of registering to another municipality before December 31, 2009, or b) December 31, 2009. The dates of death were cross-checked with the corresponding death certificates of each individual, which were obtained from the local vital statistics registry and from burial records of the local church. The cause of death was coded using the four-digit ICD-9 classification system by a physician (P.K.). For the few (eight) persons who had changed municipality and then re-registered in the Oinofita municipality during the study period, we counted only the person years during which they were formally registered in the municipality.

### Statistical analysis

We calculated person years, stratified by gender, age (in five year groups), and calendar year. We also calculated observed deaths for all causes, overall cancer, and site specific cancers, stratified by gender, age, and calendar year.

The expected number of deaths was calculated based on mortality statistics of the entire Voiotia prefecture, in which Oinofita municipality belongs to. Voiotia prefecture includes 20 municipalities, and had an average population size of approximately 125,000 during the years of interest. We chose Voiotia prefecture, because of the similar geographical, population density, socioeconomic, and ethnic origin characteristics of the population.

The population statistics for Voiotia, as well as the cause specific deaths (coded from original death certificates), stratified by gender, age, and calendar year, were provided by the Hellenic Statistical Authority. Hence, we were able to calculate the corresponding all cause and cause specific mortality rates by gender, age, and calendar year for the Voiotia prefecture.

Standardized Mortality Ratios (SMRs) were computed, stratified by age (in five-year age groups), gender and calendar year, by dividing the observed number of deaths with the expected number of deaths (multiplied by 100). The expected deaths were obtained by multiplying the corresponding person years with the age-gender-year and cause specific mortality rate of the Voiotia prefecture population. In the analysis, we reduced the four-digit ICD-9 code to a three-digit level classification system similar to what is used by the Hellenic Statistical Authority [19]. We used SPSS 17 and confirmed results using Stata 10. We calculated 95% confidence intervals and p-values for the SMRs on the basis of the exact Poisson method [20,21].

Tests of linear trend were performed (under the Chi-square distribution) [22], after computing cause specific SMRs (adjusted for age and gender) for each year of follow up. The reason was to use years of follow up as a proxy to exposure level (dose). Thus a linear trend (if existing) would be in accordance with a dose/response relationship taking into account latency period as well. Although municipality records do not specify the village in which each individual resides, we obtained this information from the death certificates. Thus, we were able to calculate proportional mortality ratios (PMRs) (adjusted for age and gender) of cause specific deaths versus all deaths, for specific Oinofita villages compared to those of the Voiotia prefecture.

## Results

The number of total deaths, cancer deaths, and persons years (total and within each age group), stratified by gender and calendar year are presented in Table 2. More than 50% of the person years calculated corresponded to age groups under 40, while the percentages of male and female did not substantially differ. The person years were decreasing as a function of time, showing 5232 and 4632 person years in 2000 and 2009, respectively. Thus, the rate at which persons exit the cohort surpasses the corresponding entry rate. Furthermore, it is interesting to note that in 2009 there were more cancer deaths than ever before (18 compared to 4-13 as observed in previous years).

**Table 2:** Total deaths, Cancer deaths and Person years age distribution, stratified by gender and calendar year

Year	1999	2000	2001	2002	2003	2004	2005	2006	2007	2008	2009	Total
Age distribution (% Person years)	TOTAL											

0-19	22.5	22.7	21.9	21.5	20.9	20.2	19.8	19.2	19.2	19.0	19.3	20.6

20-39	31.9	31.7	31.8	31.9	32.0	32.4	32.2	32.1	31.2	31.1	29.8	31.7

40-59	26.2	25.8	26.0	25.7	26.0	25.7	25.6	25.3	25.4	25.3	25.6	25.7

60-79	16.7	17.1	17.6	18.4	18.6	19.1	19.5	20.2	20.6	20.9	21.4	19.1

>80	2.7	2.7	2.6	2.4	2.4	2.7	2.8	3.3	3.7	3.7	3.8	3.0

Person years	5109.5	5232.2	5213.6	5148.0	5081.8	5037.7	4979.4	4871.2	4780.1	4717.0	4632.5	54,803.1

Total deaths	36	40	47	47	36	47	39	34	52	44	52	474

Cancer deaths	4	13	7	12	9	9	12	11	11	12	18	118

Age distribution (% Person years)	MALE											

0-19	22.7	23.6	22.7	22.1	21.7	21.1	20.6	20.3	20.1	19.7	20.0	21.4

20-39	32.0	31.2	31.3	32.0	32.1	32.5	32.4	32.2	31.4	31.6	30.7	31.8

40-59	26.1	25.9	25.8	25.6	25.9	25.3	25.5	24.8	25.2	25.1	25.5	25.5

60-79	17.4	17.6	18.2	18.6	18.7	19.1	19.2	20.0	20.1	20.2	20.2	19.0

>80	1.9	1.8	1.8	1.7	1.6	2.1	2.2	2.7	3.1	3.4	3.6	2.3

Person years	2531.5	2597.8	2587.7	2554.2	2527.8	2513.1	2481.4	2421.4	2369.5	2341.1	2295.6	27.221.0

Total deaths	19	24	29	27	17	22	26	20	27	27	29	267

Cancer deaths	2	8	7	8	6	3	10	8	5	8	11	76

Age distribution (% Person years)	FEMALE											

0-19	22.3	21.8	21.1	21.0	20.1	19.3	19.0	18.2	18.3	18.3	18.7	19.9

20-39	31.9	32.3	32.3	31.9	32.0	32.3	31.9	31.9	31.0	30.6	29.0	31.6

40-59	26.4	25.6	26.1	25.7	26.2	26.0	25.8	25.8	25.6	25.4	25.6	25.8

60-79	16.0	16.7	17.0	18.3	18.5	19.2	19.8	20.3	21.0	21.7	22.6	19.1

>80	3.5	3.5	3.4	3.2	3.3	3.3	3.4	3.8	4.2	4.1	4.1	3.6

Person years	2578.0	2634.4	2625.9	2593.8	2554.0	2524.6	2498.0	2449.8	2410.6	2375.9	2336.9	27,582.0

Total deaths	17	16	18	20	19	25	13	14	25	17	23	207

Cancer deaths	2	5	0	4	3	6	2	3	6	4	7	42

A total of 474 deaths were observed, of which 118 were cancer related (Table 3). These figures (i.e. one in four deaths being cancer related) are in accordance to the general Greek, EU15 and EU27 averages [23]. The all cause SMR for the Oinofita municipality was similar to that of the prefecture of Voiotia (SMR = 98, 95% CI 89-107). The SMR for all cancer deaths over all years was slightly increased but not statistically significantly (SMR = 114, 95% CI 94-136). There were eight observed deaths of the hepatobiliary system, and more specifically: six primary liver cancers, one bile duct, and one gallbladder. For primary liver cancer, the observed deaths were eleven fold higher than the expected number of deaths (SMR 1104, 95% CI 405-2403, p < 0.001); statistically significant SMRs for primary liver cancer were observed among both males and females. Observed deaths associated with kidney and other genitourinary organ cancers (five deaths with ICD-9 code 189, and one death with ICD-9 code 184) were more than threefold higher than expected in women (SMR 368, 95% CI 119-858, p = 0.025). The SMR for lung cancer was also statistically significantly elevated (SMR 145, 95% CI 101-203, p = 0.047). Furthermore, elevated SMRs were noted for several other cancer sites, including cancers of lip, oral cavity and pharynx, stomach, female colon, female breast, prostate, and leukaemia, but did not reach statistical significance (Table 3).

**Table 3:** Observed deaths, SMRs with 95% CI and p-values, stratified by gender and cancer type; Oinofita vs. Voiotia

		TOTAL					MALE					FEMALE				
		
Cause of death	ICD-9 range	**Obs**.	SMR	95% CI		p-value	**Obs**.	SMR	95% CI		p-value	**Obs**.	SMR	95% CI		p-value
Total deaths		474	97.9	89.3	107.1	0.661	267	108.0	95.5	121.8	0.219	207	87.3	75.8	100.0	0.502

Cancer deaths	140-208	118	113.6	94.1	136.1	0.184	76	113.6	89.5	142.2	0.293	42	113.7	81.9	153.6	0.447

Lip, oral cavity and pharynx	140-149	3	344.1	71.0	1005.7	0.117	3	468.7*	96.6	1369.6	0.055					

Stomach	151	6	120.9	44.4	263.2	0.755	4	115.6	31.5	296.1	0.909	2	133.1	16.1	480.8	0.886

Colon	153	6	83.5	30.6	181.7	0.844	1	27.7	0.7	154.3	0.249	5	139.8	45.4	326.2	0.578

Liver primary	155.0	6	1104.2**	405.2	2403.3	<0.001	4	811.7**	221.2	2078.3	0.003	2	3952.3**	478.6	14277.0	0.002

Pancreas	157	6	85.0	31.2	185.0	0.882	4	87.9	24.0	225.1	1.000	2	79.7	9.7	288.0	1.000

Gallbladder and other digestive organs and peritoneum	155.1, 155.2, 156, 158-159	4	41.6	11.3	106.5	0.075	2	32.3	3.9	116.7	0.108	2	58.3	7.1	210.8	0.669

Lung, trachea and bronchus	162	34	145.1**	100.5	202.8	0.047	29	141.9*	95.0	203.8	0.086	5	166.7	54.1	389.1	0.369

Other respiratory system and intrathoracic organs	160, 163-165	2	445.1	53.9	1608.0	0.150	2	693.3*	84.0	2504.3	0.069					

Bone and articular cartilage	170	1	128.6	3.3	716.7	1.000	1	193.0	4.9	1075.4	0.809					

Malignant melanoma of skin	172	1	143.6	3.6	800.1	1.000	1	214.9	5.4	1197.2	0.744					

Female breast	174	9	133.6	61.1	253.5	0.475						9	133.6	61.1	253.5	0.475

Cervix uteri	180	1	412.1	10.4	2296.1	0.431						1	412.1	10.4	2296.1	0.431

Prostate	185	7	127.9	51.4	263.5	0.620	7	127.9	51.4	263.5	0.620					

Testis	186	1	2141.5*	54.2	11931.5	0.091	1	2141.5*	54.2	11931.5	0.091					

Bladder	188	3	82.1	16.9	240.1	1.000	2	65.4	7.9	236.3	0.821	1	168.3	4.3	937.8	0.896

Kidney and other genitourinary organs	184, 187, 189	6	203.5	74.7	442.9	0.158	1	62.9	1.6	350.6	1.000	5	367.8**	119.4	858.3	0.025

Brain	191	4	89.4	24.4	229.0	1.000	3	107.2	22.1	313.3	1.000	1	59.8	1.5	332.9	1.000

Other and unspecified malignant neoplasm	190, 192-199	9	88.0	40.2	167.0	0.859	4	71.1	19.4	182.1	0.677	5	108.5	35.2	253.3	0.976

Leukaemias	204-208	7	167.6	67.4	345.4	0.260	5	172.8	56.1	403.4	0.334	2	155.8	18.9	562.9	0.735

Other lymphoid and hematopoietic tissue	200, 202-203	2	82.2	10.0	297.1	1.000	2	182.8	22.1	660.2	0.598					

Notes: * Statistically significant at 10% level. ** Statistically significant at 5% level. Types of cancer are classified according to the Hellenic Statistical Authority [17].

**Figure 2:**
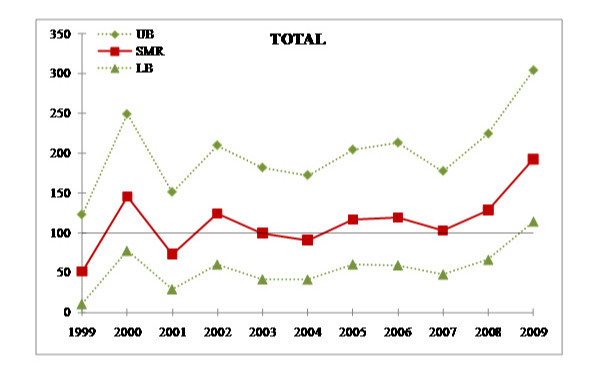
**SMRs (with 95% CI) for all cancer deaths by calendar year**.

Tests for linear trend performed after grouping the period specific SMRs into 3 time intervals, i.e. 1999-2002, 2003-2006, 2007-2009, did not reveal any significant evidence of a linear trend. However, as depicted in Figure 2, there was a statistically significant SMR of 193 (95% CI 114-304, p = 0.015) for all cancer deaths that was found for the year 2009.

Further, we noted that three out of the six deaths associated with primary liver cancer and two out of the five deaths associated with female kidney and other genitourinary organ cancers came from the small village of Agios Thomas (with only 1090 legally registered permanent residents, according to the 2001 population census). The PMR for primary liver cancer for Agios Thomas compared to Voiotia prefecture was equal to 1629 (95% CI 336-4762, p = 0.002), while the PMR for female kidney and other genitourinary organs cancer was equal to 442 (95% CI 53-1595, p = 0.153). Of interest, in measurements made by the Institute of Geology and Mineral Exploration in 2008, the highest concentration of hexavalent chromium (156 μg/l) was found in a well close to the village of Agios Thomas.

## Discussion

There is considerable debate on the potential health effects of oral exposure to Cr(VI). Based on available evidence [7-11] the California Environmental Protection Agency [24] decided to establish a criterion of 0.2 μg/l for Cr(VI) as the maximum level in drinking water in 1999. However, this criterion was withdrawn in 2001 after reviewing the weight-of-evidence [4], and concluding that there was insufficient data to consider Cr(VI) a carcinogen via the oral route. Given the absence of adequate evidence, the California Congressional Delegation, California Environmental Protection Agency, and California Department of Health Services nominated Cr(VI) for toxicity and carcinogenicity testing to the National Toxicology Program (NTP). The NTP results published recently [6] report that rats and mice exposed to drinking water with Cr(VI) developed oral cavity neoplasms, small intestine neoplasms and hyperplasia, and displayed a significant increase in histiocytic cell infiltration in the duodenum, jejunum, and liver as well as in mesenteric and pancreatic lymph nodes. This evidence contributed to the proposal of the California Environmental Protection Agency to set a public health goal (0.06 μg/l) for Cr(VI) in drinking water in August 2009 [16]. Hexavalent chromium was further recognized as 'likely to be carcinogen to humans' recently and the cancer potency to humans was estimated to be equal to 0.5 (mg/kg/day)^-1 ^(Stern, 2010).

The contamination of drinking water in Oinofita gave us the opportunity to evaluate the potential effects of hexavalent chromium on mortality rates. Though based on small numbers, our data raise concerns as to the possibility of higher mortality rates from primary liver and lung cancers in both males and females as well as urologic cancers among women, but undoubtedly further research in the area is warranted. Comparisons between genders are hard given the small numbers of observed and expected numbers of deaths, thus partly explaining the non consistent finding of increased urologic cancers only among female. Further, our results suggest possibly higher risk of other epithelial and gastrointestinal cancers. These findings are consistent with previous epidemiological and animal studies indicating carcinogenesis after consumption of drinking water contaminated with Cr(VI) [6-11].

Our study, similar to previous epidemiologic studies, was based on an ecologic comparison. Thus, exposure is expressed as residing in the area assuming that all residents consumed water provided by the municipality. Well water is very rarely used for drinking in Greece and the level of contamination is very similar. Another water source could be bottled water thus leading to lower actual exposure and therefore underestimation of the risk. Furthermore, it is not possible to exclude the presence of confounding factors such as occupational exposures and cigarette smoking. Indeed, these factors could account for the modestly increased SMRs for lung cancer in the region; however, one would have expected all causes of death to be elevated if this were true, but this was not observed. Another potential confounder is the presence of medical conditions requiring use of anti-acids and leading to lower rate of reduction of hexavalent chromium to trivalent. We have no indications that use of anti-acids is unusual in this population. The presence of other contaminants in water is an alternative explanation. Yet, none of the available measurements revealed high concentrations in other substances. Only modest levels of arsenic were detected but not consistently (levels 0-9 μg/l in 2007, 0-22 μg/l in 2008, and 0-2 μg/l in 2009). Moreover, the elevated mortality rates for primary liver cancer are difficult to attribute fully to these other factors. An additional potential problem would be the misclassification of the cause of death e.g. attributing deaths to liver cancer or kidney cancer when the real underlying cause of death was another disease. We eliminated this potential bias by scrutinizing all death certificates and excluding all metastatic liver cancers. Similar scrutiny was not undertaken in the comparison group. Thus, we would expect that the estimated SMRs would only underestimate the real risk. We also have no reason to believe that misclassification regarding cause of death would be differential between the exposed population and the control population given that the population is of similar socioeconomic level and served by the same medical services (National Health System). So any misclassification in the cause of death would be random thus not overestimating risk.

In our methodology we allowed persons that entered the municipality after the beginning of follow up to be included in the cohort, thus including persons with very low latency period in our population. From the 5842 individuals in the cohort, 753 registered into the municipality after the start of the follow up period 1/1/1999. The majority (540) were children born between 1/1/1999 and 31/12/2009. The total additional person years (including those contributed by the children) were 4478. Among members of this sub cohort three lung cancer deaths in 2001, 2004 and 2007 (occurring in persons that entered the cohort in 1999 and 2000) were observed in the period under examination. The inclusion of this sub cohort can only have led to underestimation of the risk for all types of cancer except possibly for lung cancer.

Overall the main limitation of our study is the duration of follow-up. In our data, the overall SMRs for Oinofita appeared to rise in recent years. Thus, longer follow-up, along with better characterization of Cr(VI) exposure, by focusing on specific chromium biomarkers, would help understand whether this trend would continue.

## Conclusions

Water contaminated with hexavalent chromium has been suggested as a potential carcinogen in humans through the oral route. This study provides further evidence of this relationship. In light of the potentially widespread health implications of such contamination, further studies are critically needed to explore the possible causal link between exposure to hexavalent chromium through drinking water and cancer risk. Such evidence is needed to establish guidelines for the prevention of this form of contamination and formulate public health recommendations.

## List of abbreviations

CI: Confidence Interval; Cr(VI): Hexavalent Chromium; ICD-9: International Statistical Classification of Diseases and Related Health Problems, ninth edition; PMR: Proportional Mortality Ratio; SMR: Standardized Mortality Ratio

## Competing interests

The authors declare that they have no competing interests.

## Authors' contributions

LA conceived of the study and its design, led the data analysis and interpretation, as well as the manuscript's first draft and revisions. PA participated in the design of the study, performed the statistical analysis and drafted the manuscript. CE participated in the collection and the entry of the data. KP coded the causes of death. SM led the coordination and data collection. VA participated in the study organization and coordination, and the data collection. TE participated in the study design and the statistical analysis. CCA contributed to the statistical analysis and the review of the manuscript. MKC reviewed the background and the manuscript. KMR reviewed the methodology and the manuscript. All authors read and approved the final manuscript.
